# Uncovering the role of algal organic matter biocoating on *Navicula incerta* cell deposition and biofilm formation

**DOI:** 10.1080/21655979.2023.2252213

**Published:** 2023-09-11

**Authors:** C. Y. Tong, Siew Li Lim, Mei Xia Chua, C. J. C. Derek

**Affiliations:** School of Chemical Engineering, Engineering Campus, Universiti Sains Malaysia, Nibong Tebal, Penang, Malaysia

**Keywords:** Bio-coating, biofilm, environmental biotechnologies, extracellular polymeric substances, microalgae

## Abstract

Spontaneous natural biofilm concentrates microalgal biomass on solid supports. However, the biofilm is frequently susceptible to exfoliation upon nutrient deficiency, particularly found in aged biofilm. Therefore, this study highlights a novel biofilm cultivation technique by pre-depositing the algal organic matters from marine diatom, *Navicula incerta* onto microporous polyvinylidene fluoride membrane to further strengthen the biofilm developed. Due to the improvement in membrane surface roughness and hydrophobicity, cells adhered most abundantly to soluble extrapolymeric substances-coated (sEPS) (76×10^6^±16×10^6^ cells m^−2^), followed by bounded EPS-coated (57.67×10^6^±0.33×10^6^ cells m^−2^), internally organic matter (IOM)-coated (39.00×10^6^±5.19×10^6^ cells m^−2^), and pristine control the least (6.22×10^6^±0.77×10^6^ cells m^−2^) at 24^th^ h. Surprisingly, only bEPS-coated membrane demonstrated an increase in cell adhesion toward the end of the experiment at 72 h. The application of the bio-coating has successfully increased the rate of cell attachment by at least 45.3% upon inoculation and achieved as high as 89.9% faster attachment at 72 hours compared to the pristine control group. Soluble polysaccharides and proteins might be carried along by the cells adhering onto membranes hence resulting in a built up of EPS hydrophobicity (>70% in average on bio-coated membranes) over time as compared with pristine (control) that only recorded an average of approximately 50% hydrophobicity. Interestingly, cells grown on bio-coated membranes accumulated more internally bounded polysaccharides, though bio-coating had no discernible impact on the production of both externally and internally bounded protein. The collective findings of this study reveal the physiological alterations of microalgal biofilms cultured on bio-coated membranes.

## GRAPHICAL ABSTRACT

**Figure:**
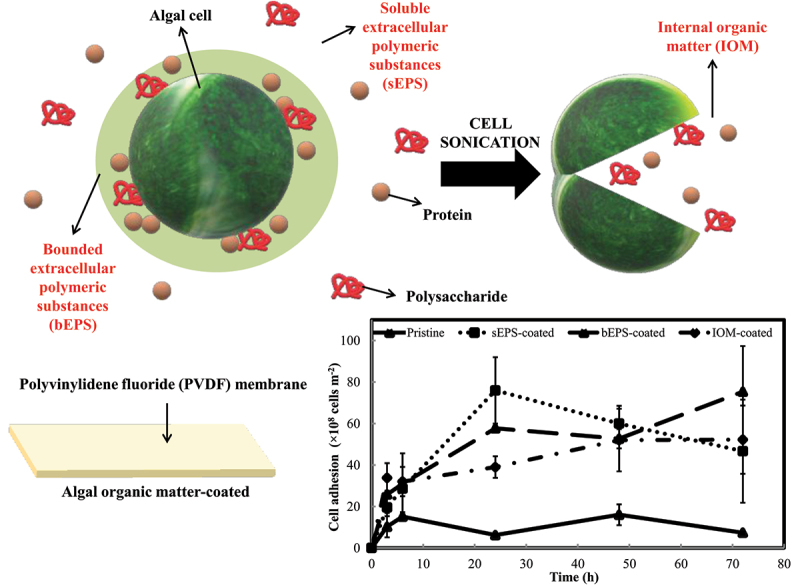

## 1. Introduction

With the escalating world population and energy demand, there is a rising need for renewable energy supply from photosynthetic microalgae which can survive in wide range of aquatic habitats and own high tolerance to harsh environments. These microorganisms are rich source of carbonaceous compounds for the production of biofuels, health supplements, cosmetics products and pharmaceutical products, as well as being a potential candidate in wastewater treatment and carbon dioxide mitigation [1]. The state-of-the-art cultivation technology used to grow these algae using immobilized biofilm on porous substrates offers huge reduction of liquid volumes by several orders of magnitude, ease in biomass harvesting, low contamination risk, and efficient separation for algal cells from the culture medium [2]. The insights gained in this biophysical process provide solutions for long-term operation of such porous substrate bioreactors; however, a main engineering challenge should be emphasized that microalgal biofilm is susceptible to exfoliation once nutrient deficiency sets in or, most of the biofilm cells start entering aging phase [3]. Cell detachment could occur by dispersal of single cell movement or sloughing off of huge cell clumps, and the situation is exacerbated at the end of natural biofilm lifecycle, eventually leading to severe biomass loss [4]. Furthermore, not all the microalgae are conducive to biofilm development and strongly attached to the selected substrates for a long period.

Hence, a number of studies have begun to examine the biological functions of bio-coatings or bio-composites on cell entrapment onto any feasible solid supports to facilitate the high performance of environmental applications. Bio-composites are typically referred as a combination of matrix material (bio-coating) with natural or synthetic fibers as reinforcement to create a multilayered porous support comprising of active microalgae which are entrapped between this coalesced adhesive latex. For instances, four different cyanobacteria strains (*Synechococcus PCC7002*, *Synechocystis PCC6803*, *Synechocystis PCC6308*, and *Anabaena PCC7120*) were used to study the concept of latex coatings of cyanobacteria with a porous paper nonwoven substrate in enhancing the photoreactivity of suspended cyanobacteria by 5- to 10-fold [5]. The bio-coatings maintain enzyme concentrations and photo-pigment activity while utilizing the biological hosts to build these biochemical systems. Colloid-based latex bio-composite was able to concentrate cells of at least 500- to 1000-fold at a high volume fraction (>50% by weight) by incorporating carbohydrate osmo-protectants to maintain the cell viability during ambient drying condition [6]. *Chlorella vulgaris* was incorporated into porous paper with addition of chitosan for carbon dioxide bio-fixation over 15 hours of operation in a solar energy–driven microbial spinning disc gas absorber-converter [7]. Nevertheless, the major challenges of these early bio-coatings were low coating porosity, poor mechanical stability, and lack of cell viability data [8].

In view of this, a simple bio-coating could also be derived from the extracellular polymeric substances (EPS) of microorganisms which are readily available and do not necessitate complicated preparation steps. Inspired by the conditioning layer phenomenon during natural biofilm development over any solid supports, this EPS bio-coating manages to sustain cell growth in thin (2-<50 μm) organic adhesive polymer coatings with minimal diffusion resistance and without any involvement of external chemically based organic glue (chitosan, glycerol, latex binder, etc.). For example, EPS-coated cotton carrier significantly improved the attached microalgae, *Scenedesmus*. LX1 density as high as 230% and protein content on an average of 15% more than uncoated carriers [9]. Rough polylactic acid sheet coated with 12-day-old *Limnothrix* sp. culture’s EPS was shown to be the best substratum to support the highest adhesion capability of around 94.60% [10]. Investigations from our former study demonstrated that cell binding affinity of three marine microalgae, namely *Amphora coffeaeformis*, *Cylindrotheca fusiformis*, and *Navicula incerta,* was 10 times greater on EPS-coated membranes with specific cell adaptation period toward the bio-coatings derived from different microalgae species [11].

The biology of growing microalgae and their reactivity when in contact with different surface chemistries are still poorly understood. Therefore, the current research is going to be an extension of our previous work on the effect of type of algal organic matter (AOM)-derived bio-coating toward the cell binding affinity of *Navicula incerta*. This particular marine diatom not only serves as a significant contributor to marine biofilms in nutrient-rich water [12], but also exhibits high metabolic sensitivity toward detectable amounts of various organic and inorganic substances [13]. Under optimal condition, it is able to accumulate high level of lipid (25–40% dry cell weight) [14,15]. Therefore, it has the potential to be applied in nutrient recovery from nutrient rich waters while generating value-added bio-compounds. This study would generate another fresh insight into the application of AOM, which primarily categorized into soluble, externally loosely bounded and internally bounded, depending on how tight the AOM are associated to the cell surfaces. Pre-treated microporous membranes were placed in a submerged biofilm cultivation system for a short-term cell adhesion experiment to assess the biochemical composition changes of the attached algal cells. This current work also checks the relative hydrophobic fraction in the AOM as increasing hydrophobicity always triggers specific interacting forces to induce irreversible cell adhesion. It aims to establish a proof-of-concept on this naturally derived bio-coating and to probe into the evolution of AOM characteristics from cells grown on AOM bio-coated substrates. Insight attained from this study could address the concern of biofilm sloughing in process intensification, including wastewater treatment, air purification, and carbon capture in future.

## 2. Materials and methods

### 2.1. Microalgae strain and cultivation

*Navicula incerta* (UTEX 2044) was purchased from the University of Texas at Austin and cultivated in sterilized 250 mL f/2 + Si medium (autoclaved at 121 \textdegreeC for 15 min). After two weeks, cultures were sub-cultivated in 1.6 L sterilized fresh medium with the aid of continuous atmospheric air bubbling at a rate of 0.2±0.1 vvm (filtered through Midisart 0.2 μm PTFE membrane filter, Sartorius, Germany) under fluorescent tubes (24.29 μmol m^−2^ s^−1^) at 25±2 °C for another two to three weeks. Average microalgal cell concentration was regularly monitored by cell counting using Neubauer chamber till approximately $1.5\times 10^{6}$ cells mL^−1^ was obtained in order to be used as the seed of subsequent adhesion experiment and algal organic matter extraction.

### 2.2. Algal organic matters extraction and pre-treatment of microporous membrane with algal extract

The algal organic matters (AOM) used in this current study were soluble extracellular polymeric substances (sEPS), bounded extracellular polymeric substances (bEPS), and internally bounded organic matters (IOM) respectively. These AOM were easily isolated from same batch of cultures according to the steps highlighted by [16]. In order to obtain sEPS, 500 mL of cultures were centrifuged at 2,504 ×g for 15 min, followed by filtering the supernatant through cellulose acetate membrane filter (47 mm diameter, Sartorius, Germany). The filtrate was collected as sEPS. Next, 500 mL of 1.5 M NaCl solution was added into all the harvested cell pellets and incubated at 30 \textdegreeC for 1 hour. Similarly, the mixture was again centrifuged and filtered at the same pre-described condition. The filtrate was then collected as bEPS. For IOM extraction, 500 mL artificial seawater (33 g of seasalt in 1 L distilled water) was used to re-suspend all the cell pellets (from bEPS isolation) and undergone sonication at 37 kHz for 30 min. Samples were again subjected to centrifugation and filtration treatment at the same operating conditions to obtain clear cell-free supernatant, known as IOM. Subsequently, these extracted AOM (Supplementary data S1) were used as the main bio-coating solution for the microporous solid supports.

The selected microporous substrate for biofilm cultivation was polyvinylidene fluoride (PVDF) membrane purchased from commercial source (Durapore 0.1 μm pore size, Merck, Ireland). This microporous membrane was selected because it has excellent thermal stability, chemical resistance, and mechanical property which make it suitable to be widely employed in wide range of water treatment industries or membrane-based photobioreactors. It was cut into 7.5 × 2.5 cm rectangular strips followed by soaking them in 10% ethanol solution for 15 min to remove the protective coatings and humectants present. Air-dried membrane strips were then pre-coated by filtering 30 mL of each bio-coating solution under negative pressure and air-dried. To ensure a uniform AOM coating onto the membranes, similar procedure was repeated three times.

### 2.3. Surface characterization of bio-coated microporous membrane supports

Each type of bio-coated PVDF membranes was characterized by surface roughness and surface hydrophobicity with pristine untreated membranes as control. Variation in membrane surface topography was checked by scanning the sample over a 20 × 20μm area using an atomic force microscope (Dimension Edge, Bruker, Billerica, MA, USA) to generate a 3D view. On the other hand, LAUDA surface analyzer LSA 200 (LaudaKönigshofen, Germany), a video-based optical contact angle measurement tool was used to compute the water angles in contact with the membrane surfaces. Membranes were evenly fixed onto glass slides during measurement and images were instantly captured within 5 seconds after 10 μL water dropping. Data was reported on average from multiple readings on different sample spots.

### 2.4. Establishment of biofilm cultivation flow lane

A self-designed flow lane incubator was adapted from our previous study with proper inlet and outlet to continuously channel high-concentrated cell suspension over the submerged pre-coated membranes for 72 hours [17]. The effective cell adhesion area per membrane was 5 × 1.5 cm. Triplicate sampling was performed at random location along the flow lane at 3^rd^, 6^th^, 24^th^, 48^th^, and 72^nd^ h.

### 2.5. Determination of adhered cell number and qualitative observation with scanning electron microscope

At each time points, samples gathered were carefully rinsed with distilled water to remove loosely bounded algal biomass from the membranes. Prior to cell harvesting, digital photos of each samples were taken to record the changes of surface coverage with biofilm over time. A minimum of three small sections cut from the cell-attached membranes were stained with approximately 1 to 1.5 mL of Alcian blue 8GX (0.01% in 0.03% acetic acid, pH 2.5) to visualize the abundance of transparent exopolymer particles (TEP). Stained samples were immediately viewed under light microscope (BX-51, Olympus, Japan) to prevent dehydration. Multiple images were captured across different membrane spots for comparison purpose, but only representative areas were presented. Remaining sections of the samples were chemically fixed in 2.5% glutaraldehyde solution, followed by washing in pH 7.2 phosphate buffer solution and gradual replacement in series of ethanol concentrations (35%, 50%, 75%, and 95% for 10 min each). Next, dehydrated samples were cryogenically fractured in liquid nitrogen to attain sharp cross sections without significant artifacts. Using a scanning electron microscope (Quanta 450 FEI, Hillsboro, OR, USA) at 5 kV acceleration voltages, cross sections’ images were acquired for biofilm thickness quantification by ImageJ analysis software (Rasband 1997–2011). Similarly, a minimum of three sections were viewed under SEM and measurement of biofilm thickness was performed along the cross sections acquired with ImageJ. Final biofilm thickness values were determined from the mean of five random sampling points measured per each section and then further averaged for triplicates. Adhered microalgal cells were eventually harvested from triplicate membranes by sharp flat tool to be re-suspended in 5 mL of 1.5 M NaCl for cell counting purpose.

### 2.6. Composition analysis and hydrophobicity fractionation of algal organic matters

sEPS was collected from free-flowing medium in the flow lane whereas similar isolation protocols for those from bEPS and IOM in Section 2.2 were applied in the retrieved samples from the biofilm cultivation flow lane. Total polysaccharide and protein contents of those AOM solutions were quantified employing phenol-sulfuric acid method [18] and BCA protein assay kit (Novagen, Merck, Germany). Colorimetric analysis was performed with a UV-Vis spectrophotometer (Cary 60, Agilent Technologies, USA) at wavelength 490 nm and 562 nm respectively. In terms of AOM hydrophobicity, AOM were further fractionated into hydrophobic components using DAX-8 resins which adsorb hydrophobic compounds in a Spin-X centrifuge tube filter (0.45 μm cellulose acetate, Corning, United States). The polysaccharide and protein content before and after adding XAD-8 resin was measured in accordance with the methods outlined above. The percentage decrease in content is their relative hydrophobicity contributions and was determined employing the following equation:

$$AOM\;hydrophobicity=\frac{AOM_{before}-AOM_{after}}{AOM_{before}}\times 100\%$$

### 2.7. Statistical data analysis

By one-way analyses of variance (ANOVA), pre-coating effects were tested on the microalgal cell adhesion and AOM concentration separately at each respective time points. Being interested in the interaction between type of AOM-coated membranes and EPS profile, the significant effects were further explored by comparing all combinations using *post hoc* Tukey honest significant difference tests. All statistical analyses were performed with IBM SPSS Statistic 25 using three replicate readings for each data set input. Error bars in each figures indicated standard deviations of each averaged means from the experiments to validate the data collected.

## 3. Results and discussion

### 3.1. Effect of bio-coating on membrane surface characteristics

**Figure 1.**
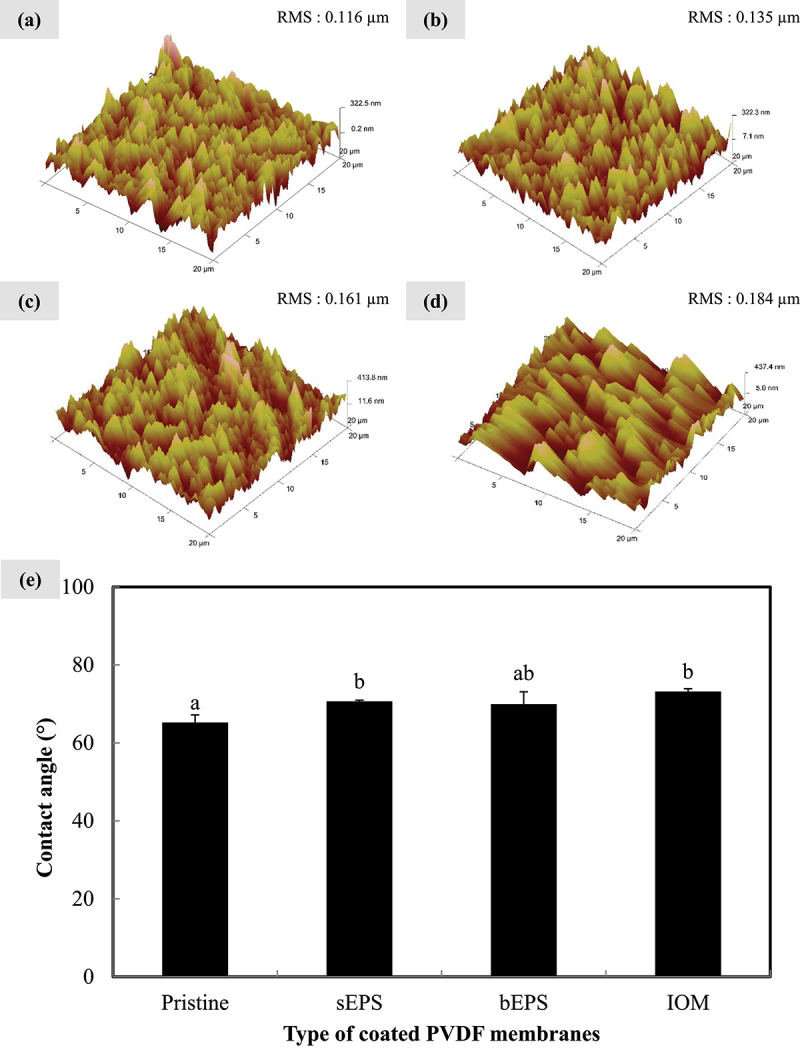
Atomic force microscopy images of (a) pristine, (b) sEPS-coated, (c), bEPS-coated, and (d) IOM-coated PVDF membranes; (e) water contact angles on each type of PVDF membranes with pristine membrane as control group. Bars represent triplicate mean± standard deviation. Different lowercase letters on the bars signify the significant difference (*p* < 0.05) according to the ANOVA analysis and post hoc comparison.

The influence of AOM coating extracted from *N. incerta* on the PVDF membrane surfaces was examined and presented in Figure 1. The membrane surface roughness was enhanced by pre-deposition of the AOM in an increasing order: pristine < sEPS-coated < bEPS-coated < IOM-coated (Figure 1(a–d)). Surface of PVDF membrane with deep valleys and high peaks, especially in Figure 1(d), was remarkably responsible for the deposition of algal organic substances or so-called foulants in membrane-based treatment processes. The Wenzel model predicts that surface hydrophobicity would increase with surface roughness and the results as displayed in Figure 1(e) were in an agreement with this theory [19]. Due to its intrinsic mildly hydrophilic characteristics, the contact angle of pristine PVDF membrane was 65.23${\;}^{\circ }\pm$1.96${\;}^{\circ }$, which was similar to a previous study [20]. Contact angles of AOM bio-coated membranes (sEPS-coated: 70.65${\;}^{\circ }\pm 0.32^{\circ }$; bEPS-coated: 69.95${\;}^{\circ }\pm$3.14${\;}^{\circ }$; IOM-coated: 73.19${\;}^{\circ }\pm$0.71${\;}^{\circ }$) were significantly higher than that of pristine PVDF, signifying a favorable role in facilitating the adsorption of hydrophobic protein-like substances and dissolved macromolecules, which are commonly known as AOM [21]. The presence of hydrophobic organic adhesins could significantly reduce the overall interfacial tension between PVDF membranes and algal cells, enabling the cells to overcome the energy barrier and adhere to the membrane surface with ease [22,23]. Moreover, during natural biofilm development, pre-deposition of AOM would serve as an effective and nutritious conditioning layer to further nourish the adhered cells, facilitating the construction of biofilm matrix [2]. In short, surface characterization results above have confirmed the presence of AOM coating onto the membrane surface.

### 3.2. Effect of bio-coating on cell adhesion profile

**Figure 2.**
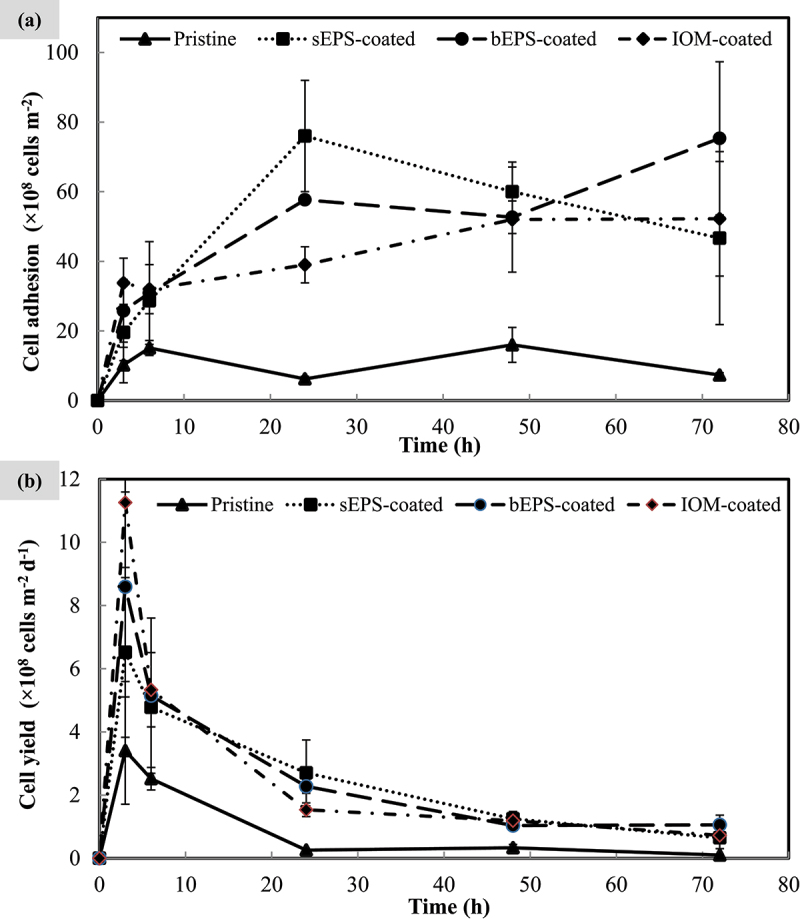
*N. incerta* (a) cell adhesion profile and (b) cell yield onto each type of bio-coated PVDF membranes with pristine membrane as control group over 72 h. Bars represent triplicate mean± standard deviation.

The adhesion performances of *N. incerta* cells on PVDF membranes prepared with different type of AOM bio-coating are shown in Figure 2(a). Apparently, cells attached on pristine membrane control was the least among all and varied less significantly across each time points (F = 5.193, *p* > 0.01). sEPS-coated PVDF membrane strips displayed the highest cell adhesion, reaching the peak of 76.00 × 10^8^ ± 16.00 × 10^8^ cells m^−2^ at 24^th^ h, but gradually decreased following that. In contrast, algal cell adhesion was found to rise consistently(F = 7.682, *p* < 0.005) on bEPS-coated membranes and achieve its highest cell colonization value about 75.33 × 10^8^ ± 22.00 × 10^8^ cells m^−2^ at 72^nd^ h. For IOM-coated membrane, cell adhesion was seemed to be reaching a plateau after 24^th^ h and varied the least (F = 2.865, *p* > 0.05) as compared with the rest. All the AOM-coated shared a similar trend for the first six hours whereby the cells began to accumulate on the membranes and exhibited their unique adhesion profiles after 24^th^ h. At the end of experiment, cell adhesion was ranked in an increasing order: pristine (7.33 × 10^8^ ± 0.67 × 10^8^ cells m^−2^), sEPS-coated (46.67 × 10^8^ ± 24.85 × 10^8^ cells m^−2^), IOM-coated (52.22 × 10^8^ ± 16.46 × 10^8^ cells m^−2^), and bEPS-coated (75.33 × 10^8^ ± 22.00 × 10^8^ cells m^−2^). Although a general declining trend in the cell yield was observed right after the third hour of inoculation (Figure 2(b)), the presence of bio-coating continued to enhance algal cell attachment throughout the entire experimental time course, as a significant difference was observed in cell yield between the pristine control and AOM-coated surfaces. Cell yield reduction was highly attributed to the perturbation from the hydrodynamic fluid of the free-flowing medium above the immobilized cells. In spite of that, Table 1 effectively illustrates the favorable impact of bio-coatings on cell attachment. The AOM bio-coating exhibited a remarkable enhancement in cell attachment rate compared to the control group. During the initial six hours, there was an approximate 50% acceleration in cell attachment speed, followed by a subsequent increase in cell adhesion rate of approximately 75% over the succeeding hours.

**Table 1.** Effects of bio-coating on cell attachment rate with pristine membrane as the main control group. Mean values from triplicates are reported, mean± standard deviation.

Average time required for different bio-coating types to achieve equivalent cell attachment as pristine control (in h)			
Pristine	sEPS-coated	bEPS-coated	IOM-coated
3	1.64±0.86	1.14±0.26	0.99±0.59
6	3.23±0.83	3.39±1.43	2.97±0.93
24	2.60±1.18	2.73±0.08	4.14±1.01
48	13.23±5.15	16.05±6.83	14.79±8.38
72	13.26±5.77	7.27±1.79	10.99±4.19

**Figure 3.**
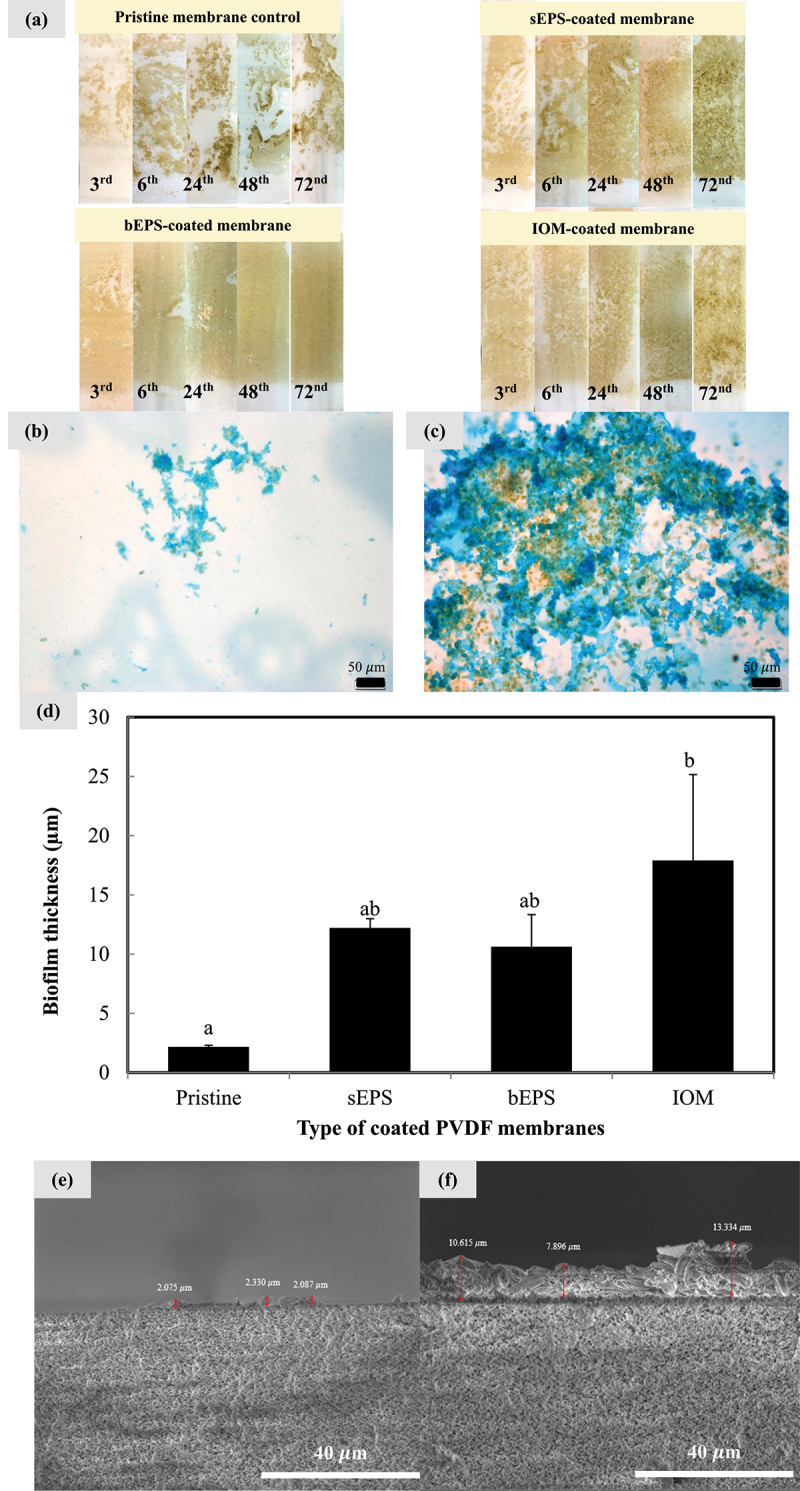
Microalgal biofilm morphology alteration and development. (a) Digital photos formed on various bio-coated PVDF membranes over 72 h; Alcian blue staining of biofilm formed on (b) pristine and (c) AOM-coated PVDF membranes at 72th h at a total magnification 200x; (d) Effect of AOM bio-coating on the biofilm thickness at 72^nd^ h; cross section images of *N. incerta* biofilm on (e) pristine control and (f) AOM-coated membrane at 72^nd^ h. Bars represent triplicate mean± standard deviation. Different lowercase letters on the bars signify the significant difference (*p* < 0.05) according to the ANOVA analysis and post hoc comparison.

As shown in Figure 3(a), it was obvious that patches of cell clumps appeared on pristine membrane control throughout 72 h. Pre-coating led to a more uniform distribution of cell coverage on the surface as compared to AOM-coated membranes, with fewer cell patches visible on the surface. Interestingly, bEPS-coated membrane showed the greatest uniformity of cell coverage across the entire experiment. Cell adhesion pattern was further assessed by Alcian blue staining. The microscopic image in Figure 3(b) revealed weaker cell adhesion on pristine membrane surface with minimal amount of transparent exopolymer particles (blue-colored region) surrounding the algal cells (brown-colored region). TEP’s gel-like properties confers it with a relatively larger viscosity than proteins, humic acids or TEP-free polysaccharides, and excellent adhesive capability onto membrane surfaces, particularly at high concentrations [24]. Comparing with the conventional organic matter mixture, TEP particles tend to demonstrate a faster adhesive rate per unit membrane area due to their high zeta potential that primarily responsible for strengthened adhesive energy, eventually giving a greater propensity for cell deposition on membrane surfaces in close proximity.Figure 3(c) shows greater abundance of TEP amount derived from the microalgae themselves on AOM-coated membranes. Cells were found closely embedded in dense TEP mat and presented extensive 2D spreading on the membrane surface, indirectly inducing the cells to aggregate and form extracellular matrix in porous 3D scaffolds [25]. A distinct difference was also observed on the TEP formation on AOM-coated membranes, which appeared as multilayered blue color intensity across the entire surfaces. Multilayer formation of TEP or an extensively extended network was observed on the samples. These TEP particles could vary from dispersed chain shape to complex cross-linked shape then transformed into dense granular shape. Previous study suggested that morphological mutation of TEP was considered a governing factor controlling the TEP-associated behavior [26]. Dense TEP as illustrated in this current work could reveal a dynamic process and capability in entrapping more cell numbers, which has coincided with the cell colonization enhancement on all the AOM-coated PVDF membranes.

Utilizing cross section technique, biofilm thicknesses on four distinct types of studied surfaces are depicted in Figure 3(d). The pristine PVDF membrane appeared to have the least biofilm thickness (2.164 ± 0.144 µm), followed by bEPS-coated PVDF membrane (10.615 ± 2.719 µm), sEPS-coated PVDF membrane (12.207 ± 0.785 µm) and the thickest biofilm formation on IOM-coated PVDF membrane (17.908 ± 7.250 µm). However, due to high degree of deviation, the differences in average biofilm thicknesses on different AOM-coated surfaces did not reach a statistically significant level. Typically, biofilm thickness being less than 20 µm is considered as young biofilms above the substratum co-localized with matrix components whereas a mature biofilm could move at a greater distance of roughly 30–40 µm from the substrate [27]. Results acquired were in parallel with the expectations made based on theory, suggesting that biofilm was still progressively developing even after 72^nd^ h until limitation of the nutrient set in. This implies that for short-term biofilm cultivation, cell adhesion dynamics will play a key role in the development and maturation of biofilm. From Figure 3(e) & 3(f), AOM-coated membranes substantially improved cell attachment whereby the cells are having a better chance to grow within the matrix internal pores and protected from fluid shear forces while cells grown on a pristine surface was more susceptible to shear and detachment. Some might argued that thicker biofilm restricted mass transfer process, but resistance to the mass transfer would be highly depending on the development of biofilm structure, especially biofilm density and porosity [28]. The prediction of diffusivity coefficient within biofilm was further complicated with non-uniform coverage of the biofilm over membranes hence it is still necessary to investigate the governing factors influencing mass transfer and determine whether biofilm thickness could be used as a simplification to describe the advection or internal diffusion in the biofilm void space.

From the observed cell adhesion trend and biofilm morphological characteristics difference above, presence of AOM bio-coating has successfully demonstrated the enhancement of cell deposition and growth profile variation which was most likely affected by a wide range of environmental conditions or perhaps, due to the cell adaptation to different types of conditioning layer [11]. In this context, it is important to further probe into their respective AOM production rates to examine the cell response or mechanisms toward the bio-coating.

### 3.3. Effect of bio-coating on overall production and hydrophobicity changes of soluble extracellular polymeric substances from *N. incerta*

**Figure 4.**
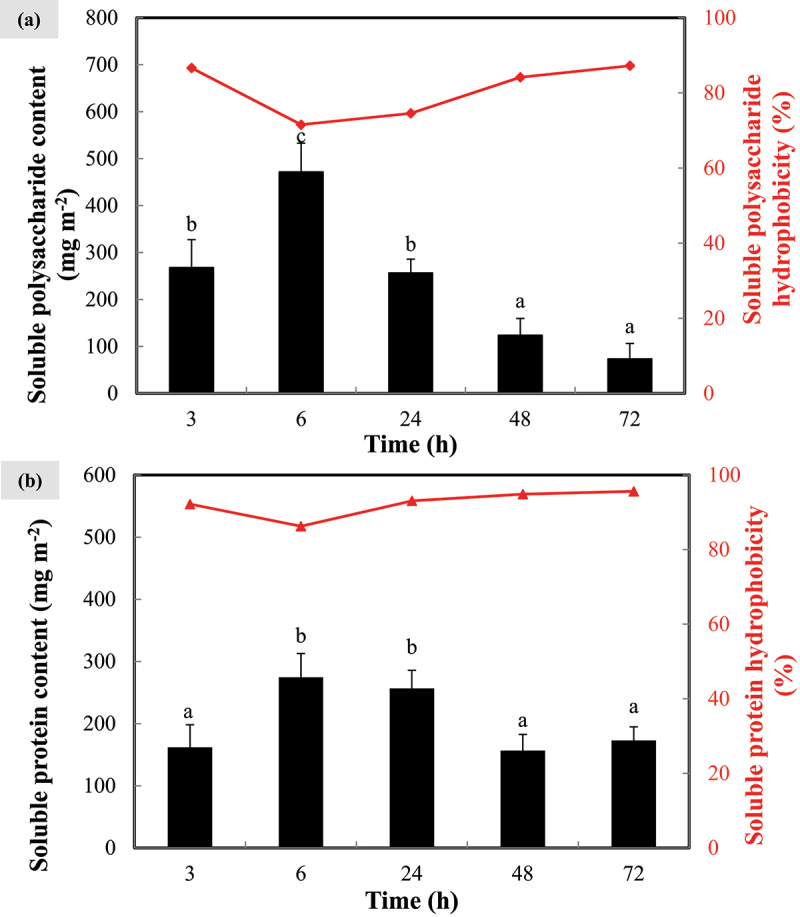
Soluble (a) extracellular polysaccharide and (b) protein content extracted from *N. incerta* cells throughout 72 h. Secondary y-axis on right hand side indicates the hydrophobicity of extracellular polymeric substances. Bars represent triplicate mean± standard deviation. Different lowercase letters on the bars signify the significant difference (*p* < 0.05) according to the ANOVA analysis and post hoc comparison across time.

As shown in Figures 4(a) & 4(b), both soluble polysaccharide and protein content displayed similar trend, which significantly increased (*p* < 0.005) during the first 6 h, followed by gradual decrease toward the end of experiment. The maximum sEPS content of polysaccharide and protein were 471.71 ± 61.26 mg m^−2^ and 274.55 ± 38.30 mg m^−2^ respectively at 6^th^ h. For the first 24 h, the concentration of soluble polysaccharide was relatively higher than that of soluble protein but it was in opposing trend after that, whereby soluble protein began to build its dominance over soluble polysaccharide. In terms of soluble EPS hydrophobicity, similar trend was also observed for both polysaccharide and protein which their respective hydrophobicity declined during the first 6 h but rose back after that. In other words, sEPS concentration was in an inverse relationship with their hydrophobicity.

It has been reported that soluble biopolymers are able to form intra- and intermolecular hydrogen bonds among EPS chains and adhesion of these biopolymers are usually weak so that they are dissolvable in solution [29]. A tremendous surge in both soluble polysaccharide and protein content was most likely attributed to active growth of free-floating cells in fresh inoculum, thereby promoting the release of EPS into the solution. Over time, cells would tend to settle down onto any available solid supports such as PVDF membrane in this study to seat themselves together and began constructing a stable conducive biofilm matrix from the surrounding nutrients. This scenario has led to the declining trend of soluble EPS after 6^th^ h, indirectly indicating the development of microalgal biofilm as soluble EPS were adsorbed as bounded form to the membranes. Adhered cells in f/2+Si medium not only consume available nutrients, but also utilize soluble EPS to increase the cohesion strength of biofilms on membrane surfaces. This is supported by the discovery that bound EPS has a strong bridging effect on cell adhesion, which contributes more to algae aggregation [30]. Similar observation was pointed out by Cui *et al*. (2022) that flocculation efficiency of microalgae *Heveochlorella* sp. Yu was highly improved from 41.47% to 59.42% by adding loosely bounded EPS [29].

It is known that soluble polysaccharide was produced more by the adhered cells compared with soluble protein because polysaccharide is regarded as the primary component of biofilm matrix that provides protection from unfavorable stress and sometimes serves as a molecular glue to tightly attach the cells to each other as well as to the solid surfaces. For instances, our previous study has proven that large amount of hydrophilic polysaccharides extracted from *Cylindrotheca fusiformis* contributed the most to biofilm development on plain drawing paper, polypropylene fabric, polyethylene plastic, and PVDF membrane while biofilm structure could also be retained by the presence of small amount of hydrophobic proteins [31]. With the EPS hydrophobicity growing over time after 6^th^ h, cells are going to benefit from this hydrophobic interaction on the membrane surfaces. Nevertheless, in view of the close association of the EPS toward the cell surfaces, bounded EPS was believed to render larger contribution on the cell adhesion.

### 3.4. Effect of bio-coating on overall production and hydrophobicity changes of both bounded extracellular and intracellular polymeric substances from *N. incerta*

**Figure 5.**
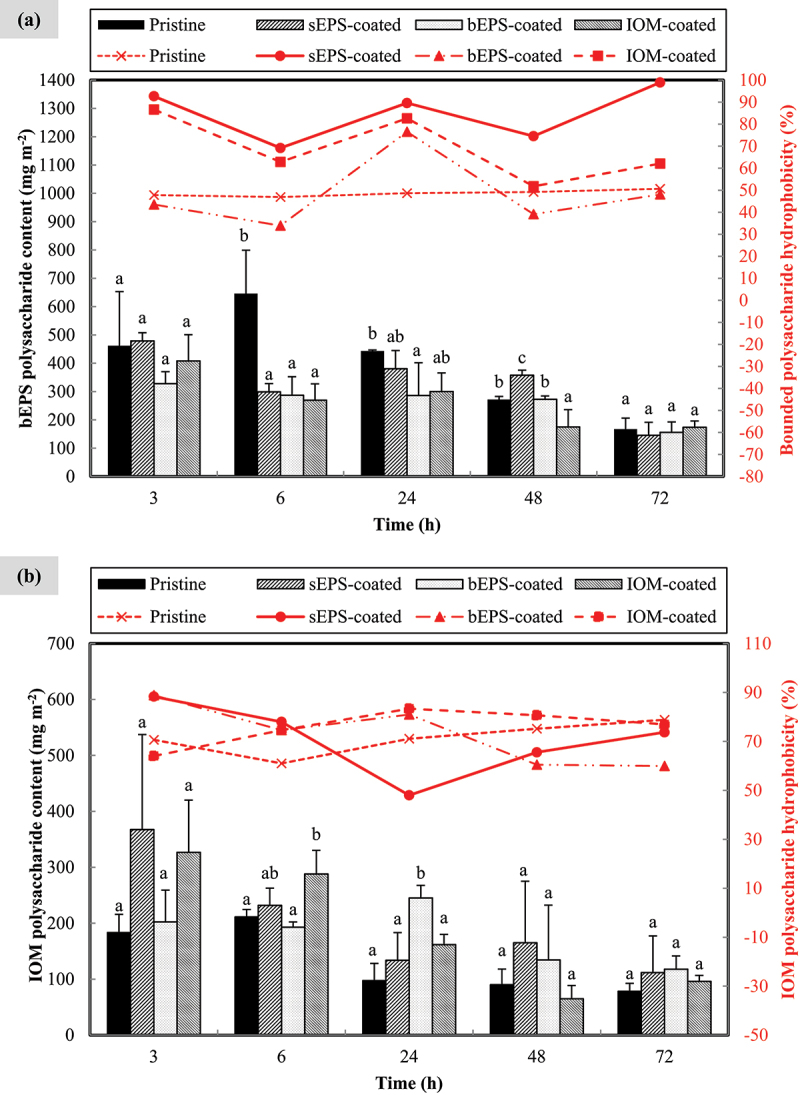
Accumulation profile of (a) bounded extracellular polysaccharide and (b) intracellular polysaccharide extracted from *N. incerta* cells adhered on different type of bio-coated membranes throughout 72 h. Secondary y-axis on right hand side indicates the hydrophobicity of algal organic matters. Bars represent triplicate mean± standard deviation. Different lowercase letters on the bars signify the significant difference (*p* < 0.05) according to the ANOVA analysis and post hoc comparison among type of pre-treated membranes at each respective time points.

As shown in Figure 5(a), the content of bounded polysaccharides recovered from cells adhered to the membranes decreased over time. The pristine control consistently exhibited the highest amount at most time points, reaching its peak value of approximately 645.99 ± 191.25 mg m^−2^ at 6^th^ h. Bounded polysaccharide content recorded by each individual group (type of bio-coated PVDF membranes) varied significantly (*p* < 0.005) across the entire experimental timeframe, with the only exception for bEPS-coated membranes (F = 2.443, *p* > 0.05). On the other hand, bEPS hydrophobicity ranged from 39.11% to 98.90% which was found to slightly increase toward the end of experiment, with the only exception for IOM-coated membrane. A noteworthy observation was bEPS polysaccharide hydrophobicity from cells adhered on pristine surface (control) was insignificantly changing throughout the time (F = 0.962, *p* > 0.10). Moreover, it was surprising to reveal that bEPS polysaccharide lost and regained hydrophobicity regularly. Overall, bEPS polysaccharide hydrophobicity could be ranked in an increasing order: pristine< bEPS-coated< IOM-coated< sEPS-coated. Conversely, profile observed for IOM polysaccharide hydrophobicity was fairly fluctuating in Figure 5(b). However, *N. incerta* cells on AOM-coated membranes accumulated considerably more (at least 1.37 times greater, *p* < 0.05) internally bounded polysaccharide than that of pristine control. Similarly, IOM polysaccharide also marked a downward trend with time, but its hydrophobicity was comparatively lower than that of bEPS polysaccharide.

**Figure 6.**
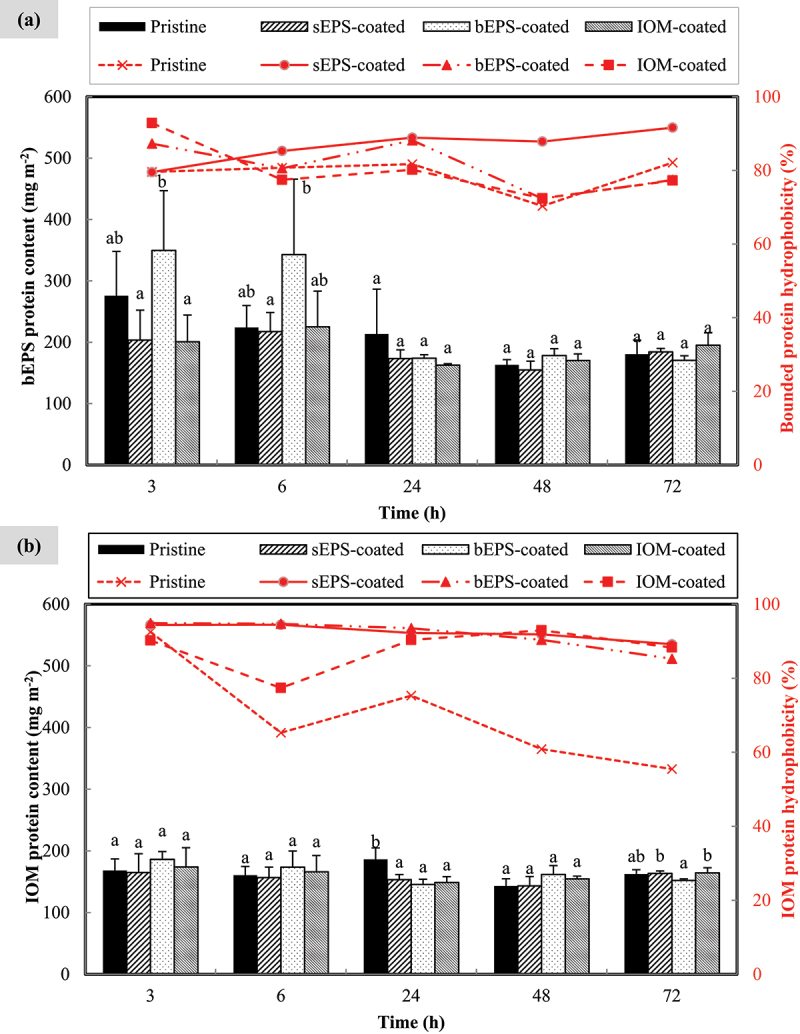
Accumulation profile of (a) bounded extracellular protein and (b) intracellular protein extracted from *N. incerta* cells adhered on different type of bio-coated membranes throughout 72 h. Secondary y-axis on right hand side indicates the hydrophobicity of algal organic matters. Bars represent triplicate mean± standard deviation. Different lowercase letters on the bars signify the significant difference (*p* < 0.05) according to the ANOVA analysis and post hoc comparison among type of pre-treated membranes at each respective time points.

On average, the overall protein content, whether externally or internally bound to other key components within the biofilm matrix, was considerably lower than the polysaccharide content. As displayed in Figure 6(a), only cells attached on pristine and bEPS-coated PVDF membranes recorded higher bounded protein content than the rest during the first 6 h. Following that, no significant difference (*p* > 0.05) was observed among all types of bio-coated membranes. In terms of hydrophobicity, bEPS protein of at least 70.33±5.61% hydrophobicity was produced but the value gradually dropped until 48^th^ h, with exception to the cells on sEPS-coated, which consistently built up their bounded protein hydrophobicity. Interestingly, as shown in Figure 6(b), IOM protein hydrophobicity determined from cells on all types of membranes decreased significantly, especially for the pristine control where it has achieved a minimum value of 55.46±7.06% at 72^nd^ h. Aside from this, no remarkable effect was found for AOM bio-coating toward internally bounded protein content as all the values were fairly consistent over time and among each individual group.

Interest was given to bEPS to understand their pivotal contribution toward cell adhesion, biofilm cohesion and growth. Indeed, recent study has reported that response of EPS production (low protein to polysaccharide ratio) was in an opposite trend with microalgal biomass, which has correlated well with the findings in this work. For example, diatom *Nitzschia palea* biofilm and green alga *Uronema confervicolum* biofilm gathered more biomass at lower EPS content and environmental disturbance enhanced both the resistance and resilience of EPS [32]. EPS has played a protective role such as *Cylindrotheca closterium* grown in xanthan gum (artificial EPS) managed to maintain cellular photosynthetic capacity though subjected to acute salinity shock treatments as high as 90 ppt [33]. Given that many researchers have demonstrated that EPS were released by cells as a metabolic response toward stressors, the current results indirectly indicated that pristine surface too is deem as environment stress thereby inducing EPS production to strengthen the cell adhesion onto such surfaces. Higher amount of bounded polysaccharides was quantified to strengthen the cell adhesion onto uncoated membranes, as well as to resist cell wash off by liquid shear force [34]. Polysaccharides bounded within the intercellular biofilm matrix undergo a transition toward increased hydrophobicity, facilitating transient binding between hydrophobic sites on the polysaccharides and hydrophobic floating cells or other species, including signaling molecules. This process ultimately promotes irreversible cell adhesion onto the membranes [17,35]. Apart from this, our previous findings have shown that bounded polysaccharides were mainly responsible for cell adhesion in *N. incerta* too [11].

Conversely, AOM-coated membranes allowed the adhered cells to accumulate more internally bounded polysaccharide and it was likely originating from the nutritious surroundings. Biofilm cells on the flat surface uptake nutrient to proliferate, hence accumulating metabolites within the cells. However, some researchers cited that excess waste metabolites released by the microalgal cell should be carried away by the medium flow to avoid product inhibition toward the cell growth [36]. To a certain extent, hyper-accumulated metabolic products would have inhibited the cell growth, chlorophyll and protein synthesis [37]. Thus, this could offer an explanation for the low abundance of internally tethered proteins that are involved in cell growth as seen in Figure 6(b). Natural biofilms typically consist of heterogeneous range of chemical constituents and physical structures, including the diversity of EPS in wide variety of chemical interactions such as ionic, hydrogen and hydrophobic interactions. The cohesive function of hydrophobic proteins has consistently been demonstrated in the formation of biofilms, as the strong hydrophobic interactions between cells facilitate the aggregation of additional free-floating cells within the dense biofilm matrix. As an example, hydrophobicity of both the extracellular and internally bounded protein from *N. incerta* cells attached on pristine membrane was among the lowest as compared to other types of bio-coating, suggesting its weakest capability to adhere a higher number of cells onto the membranes, hence giving the lowest cell adhesion.

However, the complexity within natural biofilms always makes it difficult to pinpoint the precise nature of cell-cell and cell-environment interactions, though wide spectrum of patterns could be determined [38]. The formation of stable micro-consortia, the extent of resource capture, and the retention of extracellular enzymes continue to be fundamental questions that are difficult to predict, as the extracellular polymeric substances (EPS) that mediate these processes have been characterized as the ‘dark matter’ of biofilms [39]. Furthermore, one of our former studies discovered that microalgal cells would have their own adaptation time to new bio-coating and tended to develop different mechanisms on the basis of EPS production [11]. Nevertheless, all the bio-coatings utilized in this present work were derived from the same algae species and the biofilm cultivation was for the same species back again, thereby minimizing unnecessary triggering of mechanisms against the bio-coatings. This highlights the need for further research on assessing the ability of AOM as bio-coatings and discovering its potential for promoting the growth of microalgal biofilms.

## 4. Conclusion

The underlying idea of this study is that bio-coatings derived from both extra- and intracellular polymeric substances of microalgae could render significant contribution to microalgal biofilm functional resilience. These AOM hold protective role toward the bounded cells, boosting their biomass productivities. On rougher and phobic AOM-coated PVDF membranes, *N. incerta* cells substantially adhered of at least 5 times better than that on pristine surface (control). Presence of this organic conditioning layer enhanced the uniformity of cell coverage over time, by embedding the biofilm cells within sticky boundary, which is known as TEP viscous mucilage. The findings of this study demonstrated a divergence in the polysaccharide content (bEPS and IOM), suggesting that AOM coatings of different types benefit cellular metabolites accumulation. Extracellular polysaccharides were comparably higher than extracellular protein due to its efficiency in retaining cell adhesion and formation of a protective hydrated wall that improving the biofilm resilience. Built up of AOM hydrophobicity (>70%) promoted cell adsorption and indirectly amplified the impact of the bio-coatings on biofilm formation. In future, more considerable effort should be made in anticipating the responses of biofilm components on AOM bio-coated substrates toward combined environmental stresses, as a successful establishment of stable biofilm on bio-coated substrates is crucial in real environmental applications.

## Supplementary Material

Supplemental Material

## Data Availability

The datasets generated during and/or analyzed during the current study are available from the corresponding author on reasonable request.
