# Supplementary material for: Uncovering the role of algal organic matter biocoating on Navicula incerta cell deposition and biofilm formation
Source: Bioengineered. 2023 Sep 11;14(1):2252213. doi: 10.1080/21655979.2023.2252213 (PMC10496527; doi:10.1080/21655979.2023.2252213)
Supplement: Supplemental Material [file KBIE_A_2252213_SM4816.docx]

**Uncovering the Role of Algal Organic Matter Biocoating on *Navicula incerta* Cell Deposition and Biofilm Formation**

C. Y. Tong^1^, Siew Li Lim^1^, Mei Xia Chua^1^, C. J. C. Derek^*1^

^1^School of Chemical Engineering, Engineering Campus, Universiti Sains Malaysia, 14300 Nibong Tebal, Penang, Malaysia

*Corresponding author:

Tel: +60 4-599-6414

Fax: +60 4-599-6908

E-mail address: [chderekchan@usm.my](mailto:chderekchan@usm.my)

**Fig. S1** Compositional analysis of algal organic matter solution (bio-coating) from microalgae *N. incerta*. Data are presented as the average of triplicates.
